# Stroke-associated pneumonia with low PaO_2_/FiO_2_ ratio in acute large vessel occlusion after endovascular therapy: risk factors and prognosis

**DOI:** 10.3389/fneur.2025.1598156

**Published:** 2025-07-31

**Authors:** Kun Tang, Jia Li, Yucheng Wang, Zhangbao Guo, Yun Yang, Fangliang Guo, Yin Cai, Wenhua Liu

**Affiliations:** ^1^Department of Neurology, Wuhan No.1 Hospital, Wuhan, Hubei, China; ^2^Department of Health Technology and Informatics, The Hong Kong Polytechnic University, Kowloon, Hong Kong SAR, China

**Keywords:** stroke-associated pneumonia, PaO_2_/FiO_2_ ratio, ischemic stroke, endovascular therapy, risk factor, prognosis

## Abstract

**Background:**

Stroke-associated pneumonia (SAP) often occurs after ischemic stroke. A deterioration in SAP manifests itself in a decreased partial pressure oxygen (PaO_2_)/fraction of inspired oxygen (FiO_2_) ratio, indicating gas exchange dysfunction. We aimed to investigate independent predictors and outcomes of SAP with low PaO_2_/FiO_2_ ratio among patients with acute large vessel occlusion (ALVO) undergoing endovascular therapy.

**Methods:**

We retrospectively analyzed the prospective data of consecutive adult post-interventional patients with ALVO admitted to neuro-intensive care units in Wuhan No. 1 Hospital from December 2020 to December 2022. Patients developing SAP without coronavirus disease 2019 were included in this study and divided into two subgroups: PaO_2_/FiO_2_ ratio > 240 and ≤ 240. The primary outcome was favorable neuro-function at 90 days (modified Rankin Scale score of 0–2). Secondary outcomes included hospitalization days, occurrence of symptomatic intracerebral hemorrhage, and 90-day mortality. The independent risk factors and prognosis for SAP with PaO_2_/FiO_2_ ratio ≤ 240 were identified by logistic regression analyses.

**Results:**

A total of 159 subjects developing SAP were included in this study: 53 with PaO_2_/FiO_2_ ratio > 240 and 106 with ratio ≤ 240. Compared to subjects with PaO_2_/FiO_2_ ratio > 240, those with PaO_2_/FiO_2_ ratio ≤ 240 had older ages, higher baseline National Institutes of Health Stroke Scales scores, larger proportions of baseline Glasgow Coma Scale (GCS) score of 3–8 and grade of kobuta water swallow test ≥ 3, higher white blood cell (WBC) counts (all *p* values <0.05). The independent predictors for SAP with PaO_2_/FiO_2_ ratio ≤ 240 included ages (adjusted odds ratio [OR], 1.043; 95% confidential interval [CI], 1.011–1.077; *p* = 0.009), baseline GCS scores of 3–8 (adjusted OR, 2.802; 95% CI, 1.214–6.465; *p* = 0.016), and ln-transformed WBC counts after SAP diagnosis (adjusted OR, 3.977; 95% CI, 1.226–12.896; *p* = 0.021). SAP with PaO_2_/FiO_2_ ratio ≤ 240 was robustly associated with longer hospitalization days (adjusted OR, 1.074; 95% CI, 1.01–1.143; *p* = 0.024).

**Conclusion:**

SAP with PaO_2_/FiO_2_ ratio ≤ 240 is shown in significant relevance to the prolonged in-hospital stays among post-interventional patients. Older ages, baseline GCS scores of 3–8, and higher WBC counts after SAP diagnosis can independently predict the occurrence of SAP with a lower PaO_2_/FiO_2_ ratio. Further validation studies are needed.

## Introduction

Acute ischemic stroke (AIS) attributed to large vessel occlusion (LVO) burdens the global public health and economy (1). Although endovascular therapy (EVT) effectively reduces disability and death of patients with LVO (2), the occurrence of post-stroke complications due to infection can largely worsen clinical outcomes (3–5). Of note, stroke-associated pneumonia (SAP) has been the most common post-stroke infection (6, 7). The incidence of SAP varies from 8.5 to 14.3%, and rises to 28% in intensive care unit (6, 7).

The progression of SAP is featured as the obstacle to gas exchange. The partial pressure oxygen (PaO_2_)/fraction of inspired oxygen (FiO_2_) ratio is commonly employed to assess the severity of gas exchange (8). A significantly lower PaO_2_/FiO_2_ ratio was identified in association with poor prognosis among patients with lung diseases (e.g., bacterial and viral pneumonia) (9–11). As yet little is known of the clinical impact of SAP with decreased PaO_2_/FiO_2_ ratio on post-interventional patients.

In this study, we aimed to explore independent risk factors and prognosis for SAP with low PaO_2_/FiO_2_ ratio among acute stroke patients with LVO after EVT.

## Methods

This was a retrospective study of prospectively collected data from a monocentric EVT cohort of patients with LVO admitted to neuro-intensive care units in Wuhan No. 1 Hospital between December 2020 and December 2022. The Ethics Committees of Wuhan No. 1 Hospital approved this study with patient informed consent waived (No. 2022.025), and the study followed the 1975 Declaration of Helsinki (as revised in Edinburgh 2000).

### Study population

**Figure 1:**
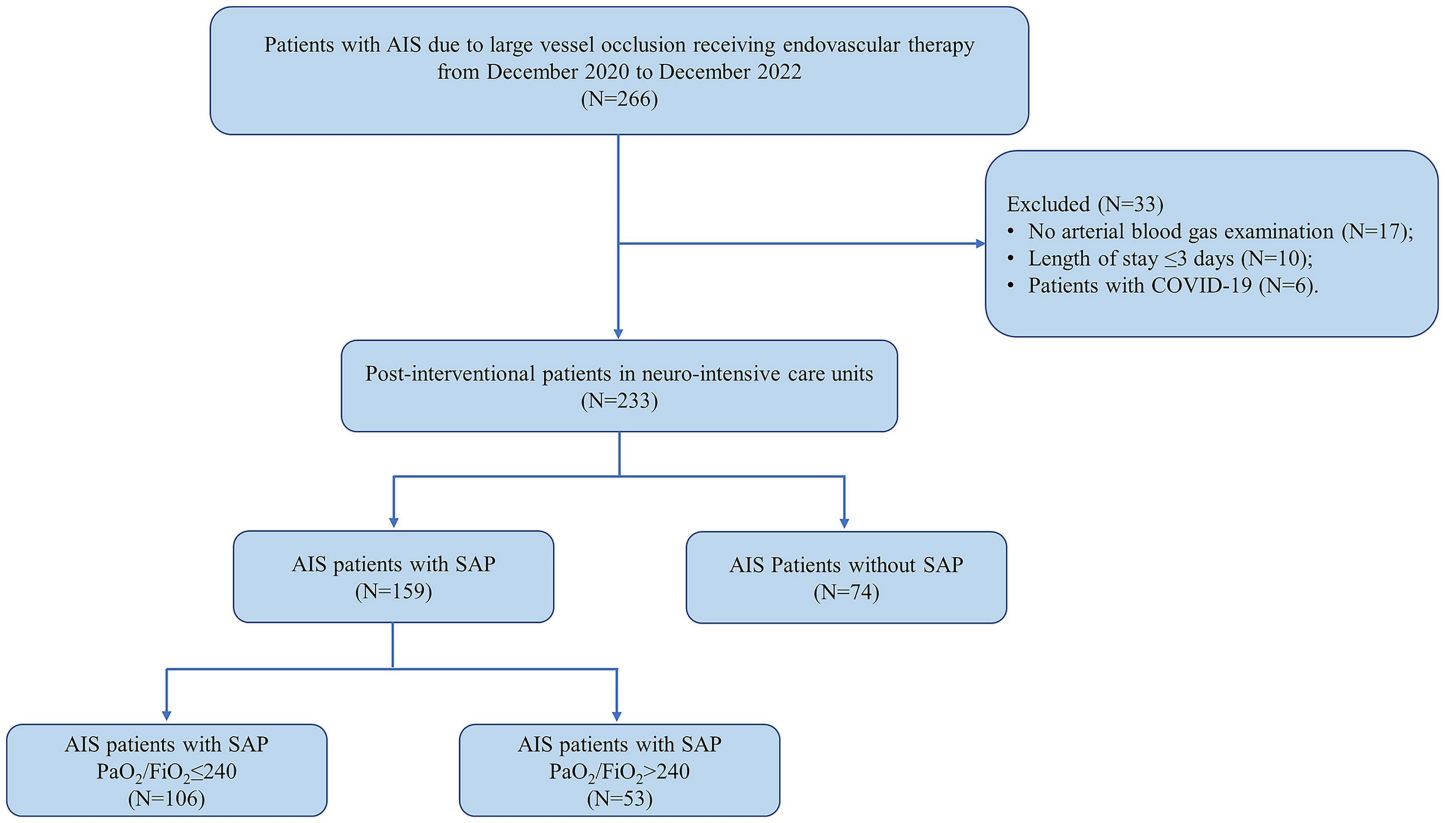
Flow diagram of patient selection in this study. AIS, acute ischemic stroke; COVID-19, coronavirus disease 2019; SAP, stroke-associated pneumonia.

In this study, post-interventional patients with SAP were included. The inclusion criteria for this study were as follows: (1) aged over 18 years old; (2) diagnosed as AIS due to LVO via angiographic modalities; (3) had a premorbid modified Rankin Scale (mRS) scored <2; (4) performed emergency EVT; (5) suffered from SAP in the first 7 days post-stroke according to the modified criteria from the Centers for Disease Control and Prevention (CDC) (The detailed diagnostic description was shown in Supplementary Table 1.) (12). The exclusion criteria included: (1) diagnosed as pneumonia before the date of new-onset stroke; (2) any contraindication to EVT; (3) diagnosed as coronavirus disease 2019 (COVID-19), based on lung computed tomography and nasopharyngeal swab; (4) any important data missing (e.g., no arterial blood gas examination); (5) length of hospitalization stay ≤ 3 days; (6) any history of immune dysfunction, malignant tumors, sepsis or other diseases that might lead to death in 3 months. The flow chart for the subject selection was presented in Figure 1.

### Data collection and follow-up

We reviewed the entire clinical parameters of all subjects from in-hospital electronic medical records (e.g., case report forms, nursing records, laboratory and radiological examinations). Patient clinical information on demographic features, past history, admission and hospitalization evaluations, stroke etiology, occlusion site, endovascular and medical therapy were collected. The worst laboratory results, including the PO_2_/FiO_2_ ratio in the most severe condition of arterial blood gas analyses and the worst white blood cell (WBC) counts, were recorded after the diagnosis of SAP. Follow-up assessments were conducted at 90 days after stroke onset via either outpatient clinics or telephone. The 90-day mRS scores were used to evaluate patient neuro-functional prognosis: a favorable function was defined as 0 to 2; death as 6. The primary outcome was favorable neuro-function at 90 days (mRS score of 0–2). Secondary outcomes included hospitalization days, in-hospital occurrence of symptomatic intracerebral hemorrhage (sICH), and 90-day all-cause mortality. sICH was diagnosed during hospitalization in line with the European Cooperative Acute Stroke Study criteria (13). The above diagnosis, disease severity and outcome evaluations were independently performed by two experienced physicians (ZB.G. and Y.Y.) in a blinded manner. In the event of any disagreement, a third physician (WH.L.) participated in discussion and made consensus.

### Statistical analysis

Statistical analyses were conducted by SPSS version 26.0 (IBM Corp., NY, United States) and GraphPad Prism version 9.0 (GraphPad Software Inc., CA, United States). Continuous variables were expressed as medians [interquartile range (IQR)], and categorical variables as numbers (percentages). According to the modified diagnostic criteria from CDC, PaO_2_/FiO_2_ ratio ≤ 240 is used as one of determinants of SAP and indicates worsened gas exchange (12), and thus PaO_2_/FiO_2_ ratio of 240 was chosen as a cut-off value in this study. The baseline clinical characteristics, therapeutic and prognostic metrics of patients with SAP were compared between PaO_2_/FiO_2_ ratio > 240 and ≤ 240 by Mann–Whitney U test, chi-square test, or Fisher’s exact test, where appropriate. Multivariate logistic regression models adjusted for variables with *p* value less than 0.05 were used to investigate the independent effect of SAP with PaO_2_/FiO_2_ ratio ≤ 240 on patient prognosis, and to further estimate independent risk factors for SAP with PaO_2_/FiO_2_ ratio ≤ 240. Logarithmic transformation (ln) was applied to WBC count to improve model fit in regression analyses via smoothing data. The diagnostic accuracy of potential predictors for SAP with PaO_2_/FiO_2_ ratio ≤ 240 was assessed by receiver operating characteristic (ROC) curve and area under ROC curve (AUC). Model calibration was evaluated via the Hosmer-Lemeshow goodness-of-fit test. A two-tailed *p* value < 0.05 was regarded as statistically significant.

## Results

### Comparisons of baseline clinical data of SAP between PaO_2_/FiO_2_ ratio > 240 and ≤ 240

One hundred and fifty-nine post-interventional patients suffering from SAP (median ages, 69.0 [59.0–76.0] years old; male, 67.3%) were included in this study: 53 with PaO_2_/FiO_2_ > 240 and 106 with PaO_2_/FiO_2_ ratio ≤ 240.

The comparisons of patient baseline clinical characters and therapeutic metrics between the two groups were listed in Table 1. Compared to subjects with PaO_2_/FiO_2_ ratio > 240, those with PaO_2_/FiO_2_ ratio ≤ 240 had older ages (70.5 [63.0–78.0] vs. 65.0 [54.5–73.0] years old, *p* = 0.003), higher baseline National Institutes of Health Stroke Scales (NHISS) scores (16 [10–21] vs. 12 [8–17], *p* = 0.005), as well as larger proportions of baseline Glasgow Coma Scale (GCS) scores of 3–8 (50.9% vs. 20.8%, *p* < 0.001) and baseline grade of kobuta water swallow test ≥ 3 (87.7% vs. 67.9%, *p* = 0.003). Besides, subjects with PaO_2_/FiO_2_ ratio ≤ 240 were more likely to have larger WBC counts (12.56 [10.64–15.83] vs. 11.36 [9.69–13.58], *p* = 0.027), after diagnosed as SAP. Yet, no statistical differences were found in other clinical features between the two subgroups (all *p* values > 0.05).

**Table 1:** Baseline clinical features and treatment metrics of SAP patients with PaO_2_/FiO_2_ ratio ≤ 240 and >240.

Characteristics	Total	PaO_2_/FiO_2_ ≤ 240	PaO_2_/FiO_2_ > 240	*p* value
	(*N* = 159)	(*N* = 106)	(*N* = 53)	
Demographic features				
Age, y, median (IQR)	69.0 (59.0–76.0)	70.5 (63.0–78.0)	65.0 (54.5–73.0)	0.003
Male, *n* (%)	107 (67.3%)	72 (67.9%)	35 (66.0%)	0.811
Medical history, *n* (%)				
Hypertension	103 (64.8%)	74 (69.8%)	29 (54.7%)	0.06
Hyperlipidemia	9 (5.7%)	6 (5.7%)	3 (5.7%)	1
Diabetes	42 (26.4%)	23 (21.7%)	19 (35.9%)	0.056
Atrial fibrillation	40 (25.2%)	29 (27.4%)	11 (20.8%)	0.366
Coronary heart disease	36 (22.6%)	23 (21.7%)	13 (24.5%)	0.688
Ischemic stroke	44 (27.7%)	30 (28.3%)	14 (26.4%)	0.802
Smoking	53 (33.3%)	33 (31.1%)	20 (37.7%)	0.405
Clinical assessments				
Baseline NHISS score, median (IQR)	15 (10–20)	16 (10–21)	12 (8–17)	0.005
Baseline GCS (3–8 scores), *n* (%)	65 (40.9%)	54 (50.9%)	11 (20.8%)	<0.001
Baseline SBP, mmHg, median (IQR)	150.0 (135.0–168.0)	152.5 (137.0–165.0)	148.0 (130.0–171.5)	0.684
Baseline DBP, mmHg, median (IQR)	83.0 (79.0–93.0)	85.0 (80.0–92.3)	82.0 (77.5–97.0)	0.971
Baseline grade of kobuta water swallow test ≥ 3, *n* (%)	129 (81.1%)	93 (87.7%)	36 (67.9%)	0.003
WBC count, 10*9/L, median (IQR)^a^	12.07 (10.25–14.88)	12.56 (10.64–15.83)	11.36 (9.69–13.58)	0.027
Lymphocyte count, 10*9/L, median (IQR)^a^	1.21 (0.81–1.82)	1.14 (0.78–2.0)	1.27 (0.90–1.72)	0.578
Neutrophil count, 10*9/L, median (IQR)^a^	6.91 (5.0–9.9)	6.91 (4.88–10.06)	6.98 (5.05–8.98)	0.698
Stroke etiology, *n* (%)				
Atherosclerotic	98 (61.6%)	67 (63.2%)	31 (58.5%)	0.564
Cardioembolic	54 (34.0%)	34 (32.1%)	20 (37.7%)	0.477
Undetermined or others	7 (4.4%)	5 (4.7%)	2 (3.8%)	1
Occlusion site, *n* (%)				
Anterior circulation	137 (86.2%)	91 (85.8%)	46 (86.8%)	0.871
Posterior circulation	22 (13.8%)	15 (14.2%)	7 (13.2%)	
Intravenous alteplase use, *n* (%)	48 (30.2%)	33 (31.1%)	15 (28.3%)	0.714
Endovascular treatment, *n* (%)				
Mechanical thrombectomy	82 (51.6%)	53 (50.0%)	29 (54.7%)	0.575
Thrombus aspiration	111 (69.8%)	75 (70.8%)	36 (67.9%)	0.714
Balloon expansion	80 (50.3%)	50 (47.2%)	30 (56.6%)	0.262
Stent implantation	57 (35.8%)	37 (34.9%)	20 (37.7%)	0.726
Time from last known well, min, median (IQR)				
To arterial puncture	300.0 (185.5–436.3)	300.0 (185.0–420.0)	339.0 (196.5–552.5)	0.234
To reperfusion or procedure completion	364.0 (269.0–531.0)	357.0 (258.0–520.0)	394.5 (272.5–610.5)	0.26
Medications in perioperative period, *n* (%)				
Antiplatelet drug	93 (58.5%)	62 (58.5%)	31 (58.5%)	1
Anticoagulant drug	65 (40.9%)	43 (40.6%)	22 (41.5%)	0.909
Statin or other lipid-lowering drug	140 (88.1%)	92 (86.8%)	48 (90.6%)	0.489
Antihypertensive drug	115 (72.3%)	80 (75.5%)	35 (66.0%)	0.21
Antidiabetic drug	17 (10.7%)	12 (11.3%)	5 (9.4%)	0.717
Antibiotic drug	159 (100%)	106 (100%)	53 (100%)	1

DBP, diastolic blood pressure; GCS, Glasgow Coma Scale; IQR, interquartile range; NHISS, National Institutes of Health Stroke Scales; NLR, neutrophil-lymphocyte ratio; SAP, stroke-associated pneumonia; SBP, systolic blood pressure; WBC, white blood cell. ^a^Worst laboratory values after diagnosed as SAP.

### Comparisons of clinical outcomes of SAP between PaO_2_/FiO_2_ ratio > 240 and ≤ 240

**Figure 2:**
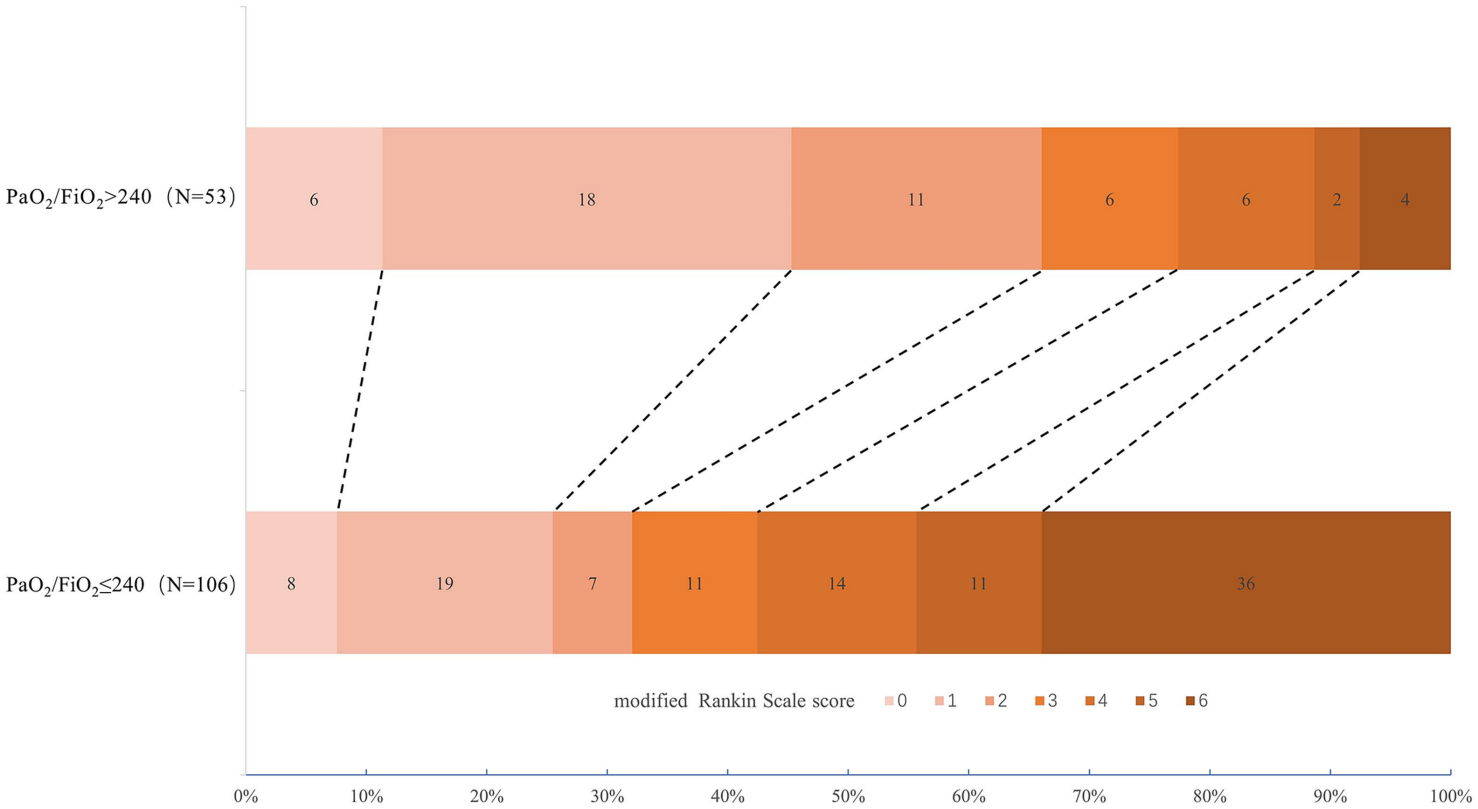
Distribution of 90-day mRS among post-interventional patients with SAP between PaO_2_/FiO_2_ ratio > 240 and ≤ 240. mRS, modified Rankin Scale; SAP, stroke-associated pneumonia.

Figure 2 exhibited the distribution of the 90-day mRS scores between the two subgroups: 66.0% of subjects with PaO_2_/FiO_2_ ratio > 240 had favorable neuro-functional prognosis at 90 days (mRS 0–2), while 32.1% of those with PaO_2_/FiO_2_ ratio ≤ 240 showed favorable 90-day neuro-function.

The comparisons of patient clinical outcomes between the two groups were displayed in Table 2. Subjects with PaO_2_/FiO_2_ ratio ≤ 240 were more possible to have a lower incidence of 90-day mRS of 0–2 (32.1% vs. 66.0%, *p* < 0.001), longer hospitalization days (13 [10–20] vs. 10 [7–13], *p* < 0.001), and a higher rate of all-cause death at 90 days (34.0% vs. 9.4%, *p* = 0.001). Yet, no statistical significance was found in the difference of sICH between the two groups (*p* = 0.111). In the multivariate analysis adjusted for ages, baseline NHISS scores, baseline GCS scores of 3–8, baseline grade of kobuta water swallow test ≥ 3, and ln-transformed WBC counts after diagnosis of SAP, SAP with PaO_2_/FiO_2_ ratio ≤ 240 was revealed in strong association with longer hospitalization days (adjusted odds ratio [OR], 1.074; 95% confidential interval [CI], 1.01–1.143; *p* = 0.024).

**Table 2:** Main clinical prognostic indicators according to PaO_2_/FiO_2_ ratio.

Parameters	PaO_2_/FiO_2_ ≤ 240	PaO_2_/FiO_2_ > 240	*p* value	OR (95%CI)^a^	*p* value^a^
	(*N* = 106)	(*N* = 53)			
mRS 0–2 at 90 days, *n* (%)	34 (32.1%)	35 (66.0%)	<0.001	0.5 (0.219–1.141)	0.1
sICH, *n* (%)	15 (14.2%)	3 (5.7%)	0.111	1.339 (0.319–5.612)	0.69
Length of stay, d, median (IQR)	13 (10–20)	10 (7–13)	<0.001	1.074 (1.01–1.143)	0.024
Mortality at 90 days, *n* (%)	36 (34.0%)	5 (9.4%)	0.001	1.702 (0.541–5.354)	0.363

CI, confidential interval; GCS, Glasgow Coma Scale; mRS, modified Rankin Scale; NHISS, National Institutes of Health Stroke Scales; OR, odds ratio; SAP, stroke-associated pneumonia; sICH, symptomatic intracerebral hemorrhage; WBC, white blood cell. ^a^Adjusted for ages, baseline NHISS scores, baseline GCS scores of 3–8, baseline grade of kobuta water swallow test ≥ 3, and ln-transformed WBC counts after diagnosis of SAP.

### Underlying risk factors for predicting SAP with PaO_2_/FiO_2_ ratio ≤ 240

Table 3 showed potential independent predictors for developing SAP with PaO_2_/FiO_2_ ratio ≤ 240. After adjusted for the confounders, the occurrence of SAP with PaO_2_/FiO_2_ ratio ≤ 240 was independently related to ages (adjusted OR, 1.043; 95% CI, 1.011–1.077; *p* = 0.009), baseline GCS scores of 3–8 (adjusted OR, 2.802; 95% CI, 1.214–6.465; *p* = 0.016), and ln-transformed WBC counts after diagnosis of SAP (adjusted OR, 3.977; 95% CI, 1.226–12.896; *p* = 0.021), rather than baseline NHISS scores and grades of kobuta water swallow test ≥3.

**Table 3:** Logistic regression analyses of potential risk factors for SAP with PaO_2_/FiO_2_ ratio ≤ 240.

Parameters	Univariate regression		Multivariate regression^a^	
	OR (95% CI)	*p* value	OR (95% CI)	*p* value
Age	1.046 (1.018–1.076)	0.001	1.043 (1.011–1.077)	0.009
NHISS score	1.076 (1.023–1.133)	0.005	1.039 (0.98–1.102)	0.198
GCS scores of 3–8	3.965 (1.844–8.523)	<0.001	2.802 (1.214–6.465)	0.016
Grade of kobuta water swallow test ≥ 3	3.378 (1.491–7.657)	0.004	1.173 (0.448–3.072)	0.746
ln-transformed WBC counts	3.787 (1.354–10.587)	0.011	3.977 (1.226–12.896)	0.021

CI, confidential interval; GCS, Glasgow Coma Scale; NHISS, National Institutes of Health Stroke Scales; OR, odds ratio; SAP, stroke-associated pneumonia; WBC, white blood cell. ^a^Adjusted for ages, baseline NHISS scores, baseline GCS scores of 3–8, baseline grade of kobuta water swallow test ≥ 3, and ln-transformed WBC counts after diagnosis of SAP.

**Figure 3:**
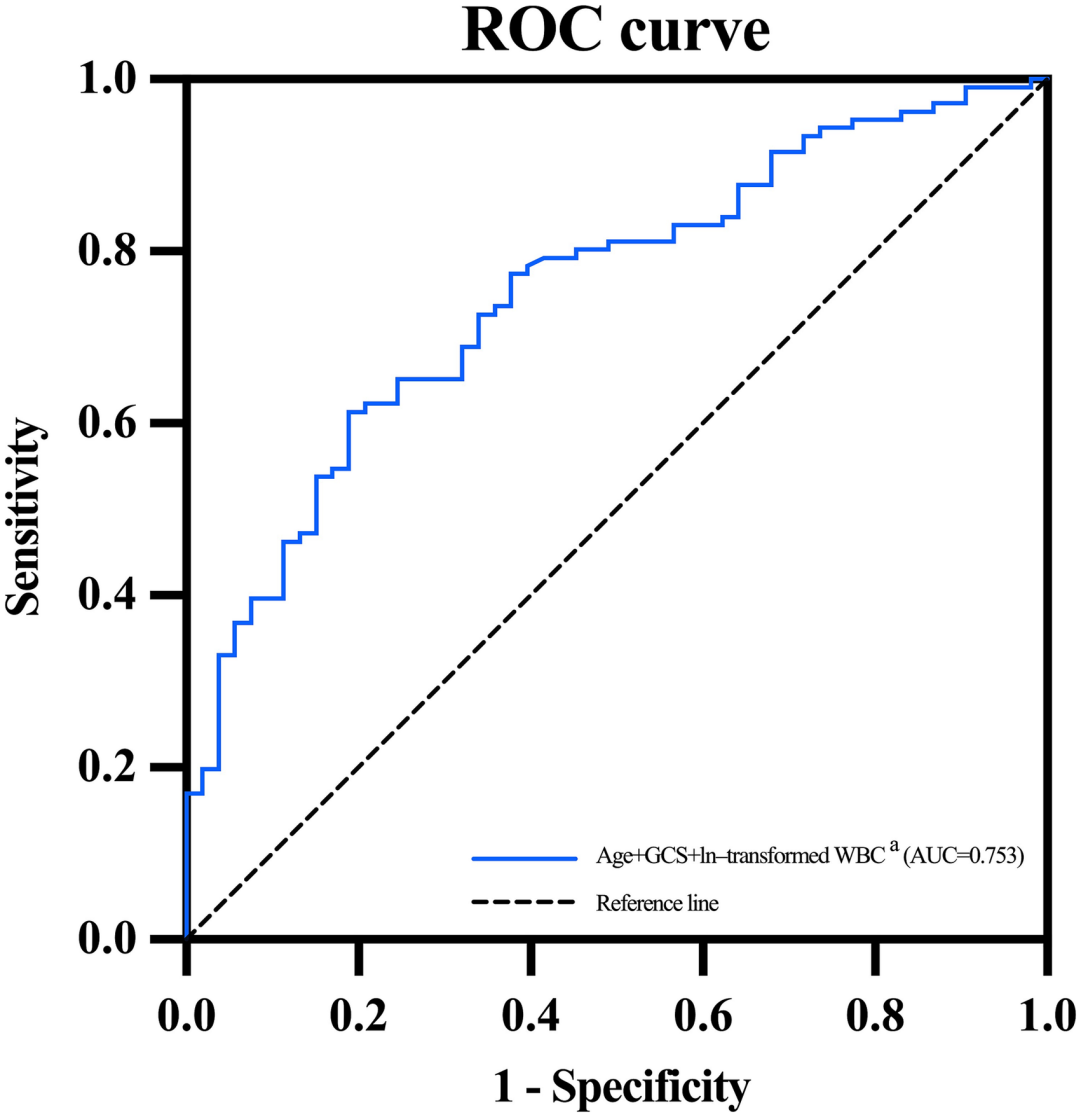
ROC curve of combined age, GCS and WBC for predicting SAP with PaO_2_/FiO_2_ ratio ≤ 240. AUC, area under the receiver operating characteristic curve; GCS, Glasgow Coma Scale; ROC, receiver operating characteristic; SAP, stroke-associated pneumonia; WBC, white blood cell. ^a^Ages, baseline GCS scores of 3–8, and ln-transformed WBC counts.

Table 4 and Figure 3 detailed the predictive effect of combined indicators (ages, baseline GCS scores of 3–8, and ln-transformed WBC counts after diagnosis of SAP) on SAP with PaO_2_/FiO_2_ ratio ≤ 240. ROC curve analysis suggested that the AUC, sensitivity, and specificity of combined indicators in predicting SAP with PaO_2_/FiO_2_ ratio ≤ 240 was 0.753 (95% CI, 0.676–0.83; *p* < 0.001), 0.613, and 0.811, respectively. The Hosmer-Lemeshow goodness-of-fit test indicated that the combined model showed a satisfactory level of goodness of fit (*p* = 0.892, χ^2^ = 3.593).

**Table 4:** AUC and diagnostic accuracy of combined age, GCS, and WBC for SAP with PaO_2_/FiO_2_ ratio ≤ 240.

Parameters	AUC	95% CI	*p*-value	Sensitivity	Specificity
Age + GCS + WBC^a^	0.753	0.676–0.83	<0.001	0.613	0.811

AUC, area under the receiver operating characteristic curve; CI, confidence interval; GCS, Glasgow Coma Scale; ROC, receiver operating characteristic; SAP, stroke-associated pneumonia; WBC, white blood cell. ^a^Ages, baseline GCS scores of 3–8, and ln-transformed WBC counts after diagnosis of SAP.

## Discussion

In the current study, firstly, we found that SAP with PaO_2_/FiO_2_ ratio ≤ 240 was robustly correlated with the prolonged hospitalization among acute stroke patients after EVT. Then, the occurrence of SAP with PaO_2_/FiO_2_ ratio ≤ 240 was revealed in independent relevance to advanced age, low GCS scores, and high WBC count after diagnosis of SAP.

Growing evidence stressed the pivotal role of SAP on the high risk of poor clinical outcomes among stroke patients (14, 15), with a large-scale study of SAP showing a 4.72-fold increase in in-hospital death related to severe stroke (16). The possible explanations could be given: First, after stroke onset, patients are predisposed to SAP due to immune dysregulation (17). SAP can also induce systemic inflammation, and thus enhance autoreactive immune responses against central nervous system antigens (18, 19). Second, SAP may have a direct and negative impact on perfusion and metabolism in the ischemic brain, especially when occurring hypotension and hypoxia (20, 21). The interaction between AIS and SAP is assumed to largely deteriorate patient conditions, thereby leading to poorer prognosis (6, 22).

Our investigation into the clinical outcomes of post-interventional patients with SAP depending on PaO_2_/FiO_2_ ratio were shown in accordance with previous observations on SAP (6, 22, 23). Notably, the PaO2/FiO2 ratio serves as a typical indicator to evaluate the severity of gas exchange in lung diseases, such as acute respiratory distress syndrome and pneumonia (24, 25). At such circumstances of lung diseases, diffuse alveolar damage may develop in lungs, then decreasing lung compliance and impairing gas-exchange function (26). We speculate that the above pathophysiological process of lung injury may also get involved in the progression of SAP among post-interventional patients, which was indicated by the decreased PaO_2_/FiO_2_ ratio.

Prior studies identified several independent predictors for occurring SAP among patients with stroke, such as existence of dysphagia, increased values of inflammatory mediators, and concurrent comorbidities (27–29). Our study further revealed that advanced age, low GCS scores, and high WBC count after diagnosis of SAP could significantly elevate the risk of developing SAP with lower PaO_2_/FiO_2_ ratio after EVT.

The underlying plausibility could be lent to our findings. Firstly, aging weakens the control of immunnometabolic responses to severe infection and persistent inflammation, while stroke-induced immunodepression can be exacerbated by inflammation-related immunosenescence during aging as well (30). Aging may thus increase the possibility of worsening post-stroke infections (30), which could be supported by our observation that older ages might increase the occurrence of SAP with lower PaO_2_/FiO_2_ ratio. Secondly, post-stroke infection and inflammation may persistently and directly damage major organ functions, including acute lung injury (31). As lung injury advances in SAP, inflammatory response escalates in lung tissue, leading to further impairment of alveolar gas-exchange function (31). Of note, high peripheral WBC count often serves as a routine and reliable indicator for systemic inflammatory status of SAP (32). Accordingly, the strong relevance of higher WBC counts to SAP with decreased PaO_2_/FiO_2_ ratio in our study may largely support the above pathophysiological process. Besides, elevated peripheral WBC counts can also independently predict extubation failure for acute stroke patients (33), which is in accordance with our finding. Thirdly, impaired consciousness attributed to stroke decreases the capacity to drive central respiratory function and airway self-clearance (34, 35). Central respiratory depression may directly result in the occurrence of hypoxemia via hypoventilation, while loss of airway self-protection may increase the risk of silent aspiration of secretions or gastric contents (34, 35). In such circumstances, gas-exchange function may easily deteriorate among patients with SAP, which can be corroborated by our result that SAP patients with low GCS were more likely to develop reduced PaO_2_/FiO_2_ ratio.

Although the combined risk factors proposed in our study showed medium diagnostic power for SAP with PaO_2_/FiO_2_ ratio ≤ 240, our findings facilitate the early identification of high-risk patients with severe SAP. Currently, no efficient therapy (neither prophylactic antibiotics nor *β*-blockers) can be approached to prevent SAP onset (36, 37). It is notable that the early prediction of SAP may largely improve overall outcomes among patients with stroke (3). Consequently, the consideration of age, baseline GCS score and WBC level after diagnosis of SAP may account for the vital impact on stroke prognosis, which still requires further validation.

Our study had limitations. Firstly, a relatively small sample size from a monocentric EVT cohort. Other unnoticed confounding factors might not be included in the models. Or combination of another relevant parameters might increase diagnostic power for severe SAP. Secondly, this study excluded patients with a confirmed diagnosis of COVID-19, which could lead to a possible selection bias, because of the fact that all patients with COVID-19 were admitted or transferred to designated hospitals during the pandemic.

## Conclusion

In this study, SAP with decreased PaO_2_/FiO_2_ ratio was revealed in significant association with the prolonged in-hospital stays among post-interventional patients with LVO. Advanced age, low GCS scores, and high WBC count after diagnosis of SAP could serve as independent risk factors for SAP with lower PaO_2_/FiO_2_ ratio after EVT. Future research is warranted to confirm our results.

## Data Availability

The raw data supporting the conclusions of this article will be made available by the authors, without undue reservation.
