# Supplementary material for: Stroke-associated pneumonia with low PaO2/FiO2 ratio in acute large vessel occlusion after endovascular therapy: risk factors and prognosis
Source: Front Neurol. 2025 Jul 31;16:1598156. doi: 10.3389/fneur.2025.1598156 (PMC12350393; doi:10.3389/fneur.2025.1598156)
Supplement: Supplementary file 1 [file Table_1.docx]

Supplemental Table. Recommended Diagnostic Criteria for SAP in Patients Not Receiving Mechanical Ventilation(12).

| At least 1 of the following: |
| --- |
| 1. Fever (>38°C) with no other recognized cause |
| 2. Leukopenia (<4000 WBC/mm^3^) or leukocytosis (>12 000 WBC/mm^3^) |
| 3. For adults ≥70 y old, altered mental status with no other recognized cause |
| And at least 2 of the following: |
| 1. New onset of purulent sputum, or change in character of sputum over a 24 h period, or increased respiratory secretions, or increased suctioning requirements |
| 2. New onset or worsening cough, or dyspnea, or tachypnea (respiratory rate>25/min) |
| 3. Rales, crackles, or bronchial breath sounds |
| 4. Worsening gas exchange (eg, O_2_ desaturation [eg, PaO_2_/FiO_2_≤240], increased oxygen requirements*) |
| And ≥2 serial chest radiographs† with at least 1 of the following: |
| New or progressive and persistent infiltrate, consolidation, or cavitation |
| Note: In patients without underlying pulmonary or cardiac disease, 1 definitive chest radiograph is acceptable |

CXR, chest x-ray; FiO_2_, fraction of inspired oxygen; PaO_2_, partial pressure oxygen; SAP, stroke-associated pneumonia; and WBC, white blood cell.

* Category of increased ventilator demand removed.

† Centers for Disease Control and Prevention recommendation is for repeat CXR at days 2±7 if initial CXR negative.
